# Supplementary material for: Cascaded emission of single photons from the biexciton in monolayered WSe2
Source: Nat Commun. 2016 Nov 10;7:13409. doi: 10.1038/ncomms13409 (PMC5109589; doi:10.1038/ncomms13409)
Supplement: Supplementary Information — Supplementary Figures 1-6, Supplementary Notes 1-3 and Supplementary References [file ncomms13409-s1.pdf]

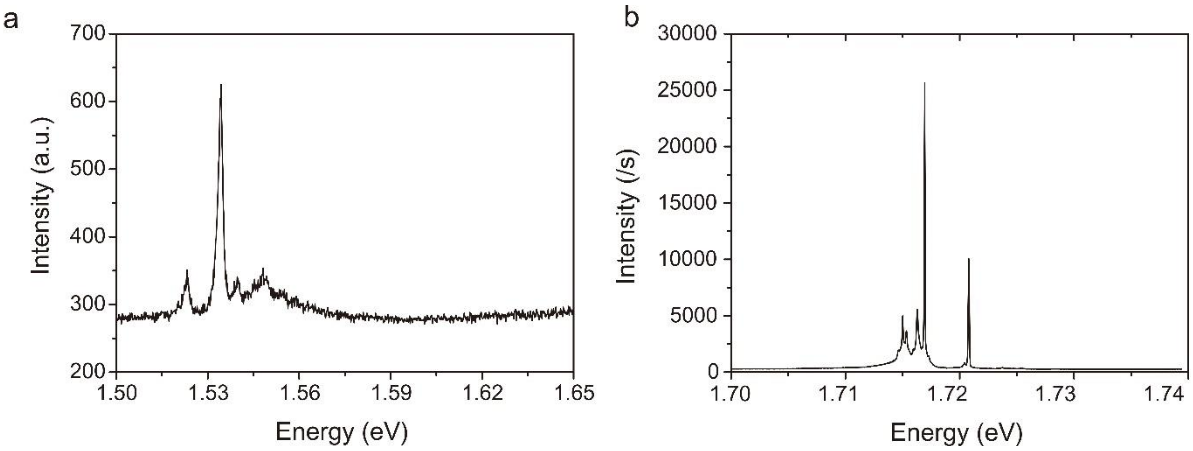


**Supplementary Figure 1| Comparison of single quantum emitters on two type of substrates:**

**a,** Photoluminescence (PL) spectrum of localized excitons in a $WSe_2$ monolayer, exfoliated onto a $SiO_2$/Si substrate (sample temperature is nominally 4.5K) . **b,** PL spectrum of localized excitons in a $WSe_2$ monolayer on a GaInP/GaAs heterostructure (sample temperature 4.5K). Here the linewidth is one order of magnitude narrower than that on the conventional $SiO_2$/Si heterostructure.

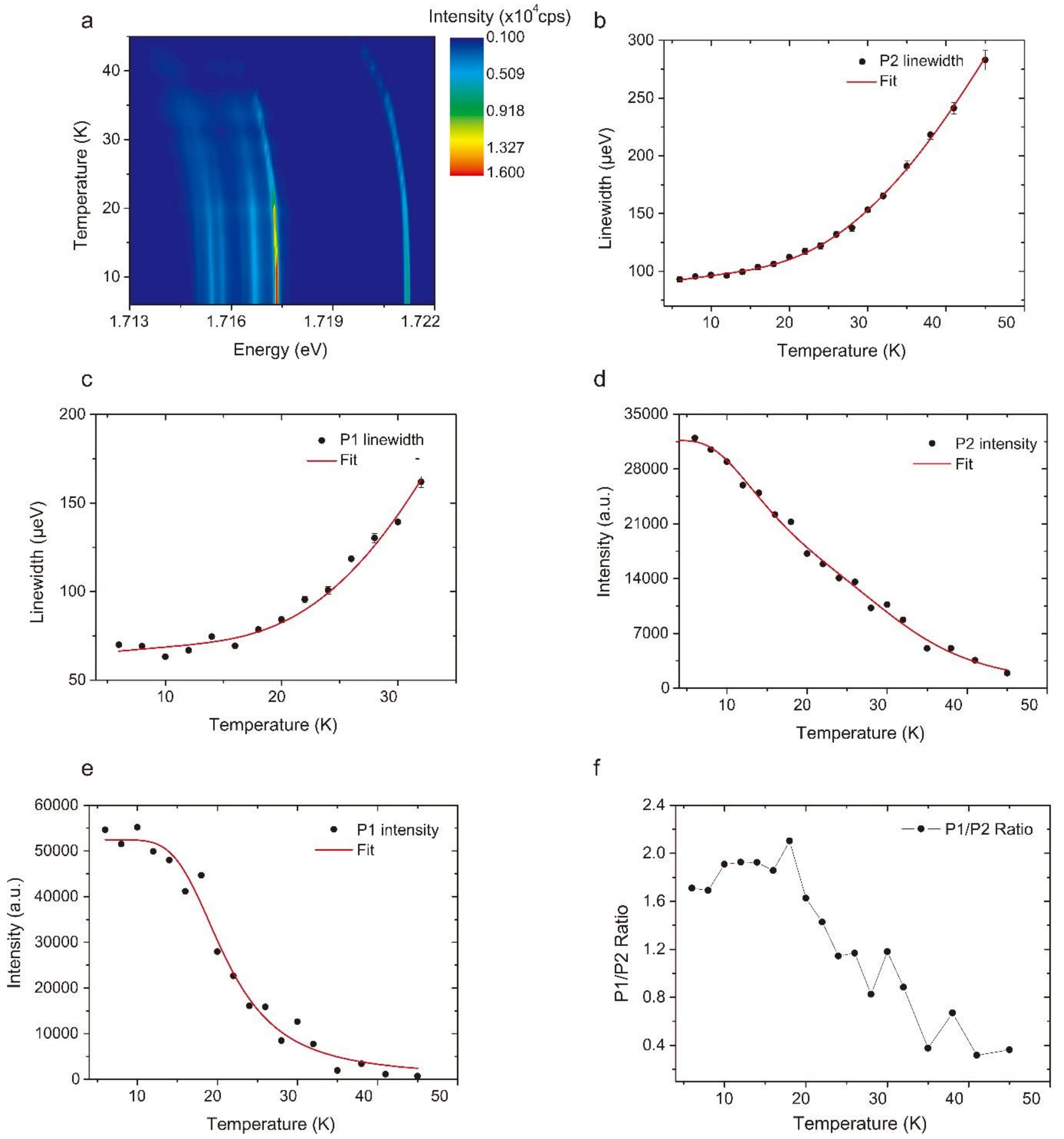


**Supplementary Figure 2 | Temperature dependence of exciton and biexciton. a,** Photoluminescence intensity plot as a function of temperature and photon energy. **b, c** linewidth of P1 and P2 in Fig. 1b in the main text. **d, e I**ntegrated counts as a function of temperature for P2 and P1. **f,** Intensity ratio between P1 and P2 as a function of temperature. The P1 intensity decreases much faster than P2 above 20K.

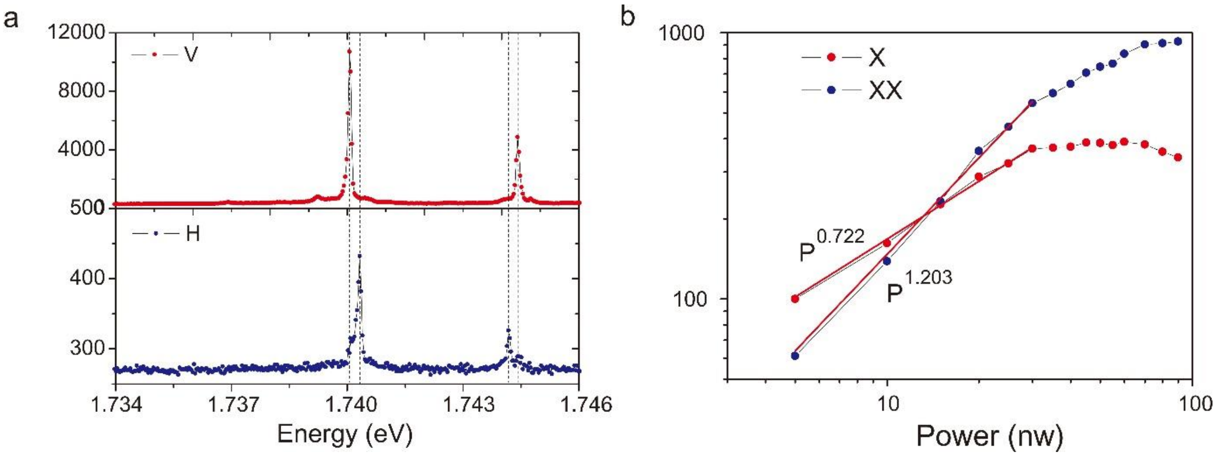


**Supplementary Figure 3 | Identification of a second correlated pair of emission lines. a,** PL spectrum of biexciton #2 is studied in the linear-polarization basis V and H. Two pairs of cross polarized spectral doublets are observed. The four dashed lines indicate the four energy positions of the X and XX attributed signal. **b,** The integrated counts of the photon emission from XX and X shows super-linear and sub-linear behavior with increasing laser power. The power law fitting yields coefficients of 0.722 and 1.203 for X and XX.

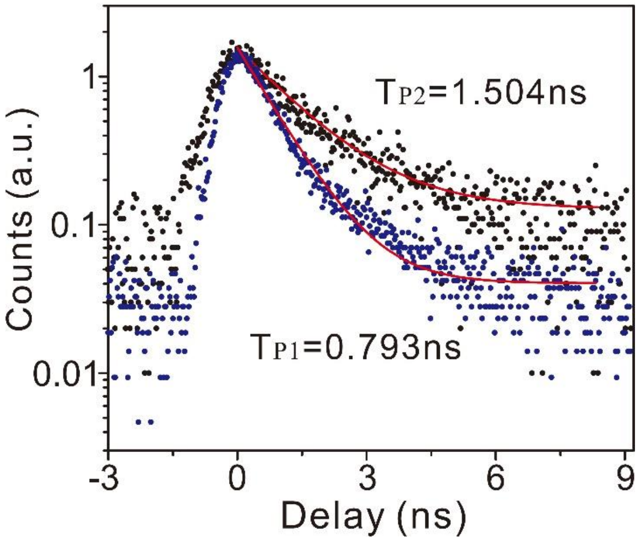


**Supplementary Figure 4| Time-resolved Photoluminescence of P1 and P2 in one laser period (12.2ns).** Compared to the XX, the X line is weak under higher excitation power and consequently it took longer time to perform the lifetime measurements. Thus the contribution of the APD (Avalanche Photon Diode) dark counts for the time resolved measurement on the X line is higher and results in an intensity offset.

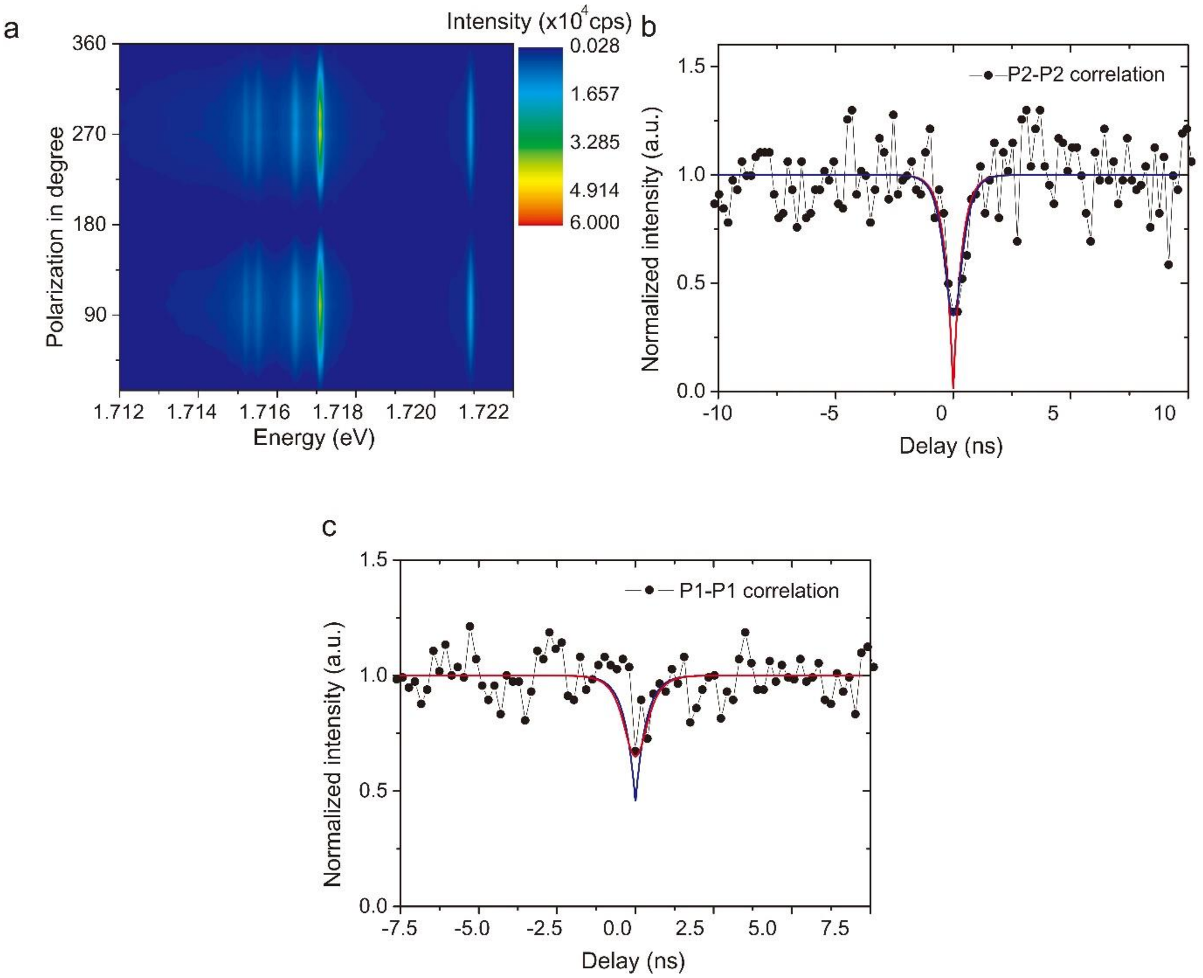


**Supplementary Figure 5 | Polarization-resolved PL and photon antibunching.**

**a,** Photoluminescence intensity plot as a function of polarization detection angle and photon energy. Compared to P1 and P2, P3 and P4 described in Fig. 2b of the main text are too weak to be directly observed. The large fine structure splitting (~0.4meV) and unbalanced emission intensity in the doublets show a significantly high level of in-plane anisotropy. **b, c** Second-order auto correlation measurement of the P2 (1.7206eV) and P1 (1.7167eV) under 70nW CW excitation at 532nm. The red lines in the pictures are the fit with according to Eqn. 5 in the main text. The blue line is the deconvoluted curve, which yields $g^2(0)$=0.014±0.002 for P2 and $g^2(0)$=0.457±0.12 for P1. Obvious photon antibunching for both lines are observed.

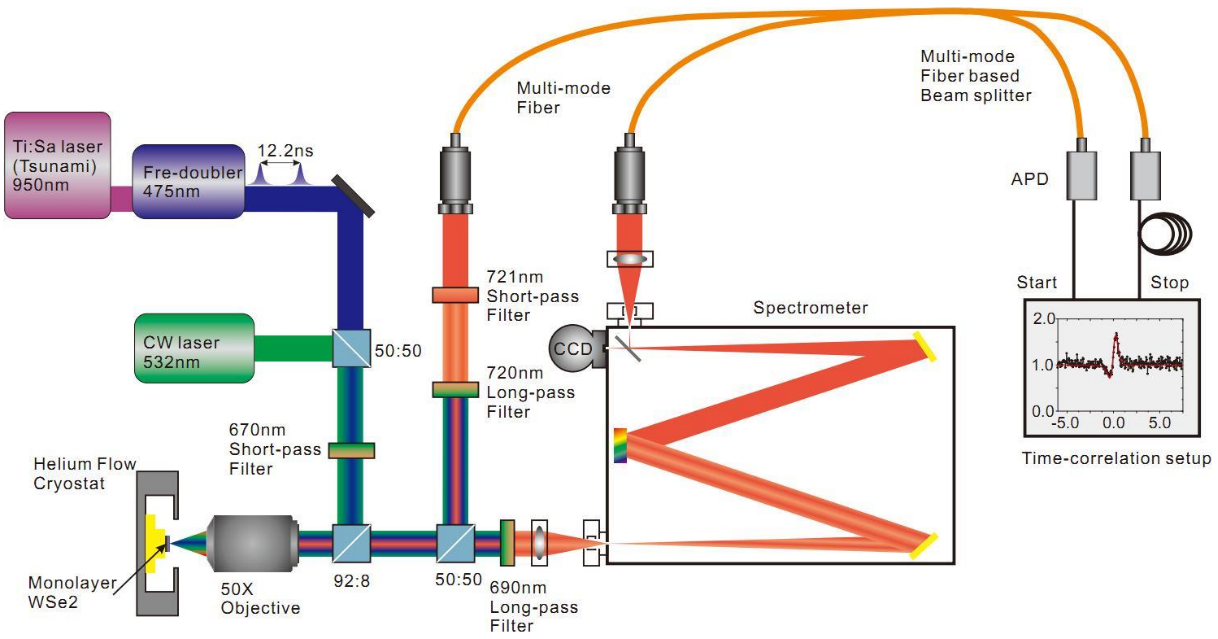


**Supplementary Figure 6 | Detailed sketch of the optical spectroscopy setup.**

## Supplementary Note 1: Comparison of single quantum emitters on two types of substrates

We believe that the successful observation of the localized biexciton in our work is a consequence of the very clean luminescence spectra, enabled by our hybrid semiconductor-monolayer heterostructure. To investigate the emission properties of the localized excitons in $WSe_2$ monolayers on different substrates, Supplementary figure 1(a) and 1(b) depict the photoluminescence from two $WSe_2$ monolayers that were transferred onto a $SiO_2$/Si and a GaInP/GaAs substrate. The observed linewidth of the monolayer on the GaInP substrate is more than one order of magnitude narrower than that on $SiO_2$ substrate (70 µeV vs. 1.9 meV). Our hypothesis is, that the semiconducting substrate allows to transfer trapped charges away from the interface, which leads to smaller spectral jitter. In addition, recent localized exciton studies on the influence of the substrate have revealed that the localized exciton linewidth and spectral wandering would be significantly enhanced after removing the $SiO_2$ substrate[1].

**Supplementary Note 2: Temperature dependent photoluminescence**

The defect's photoluminescence intensity is investigated as a function of the sample temperature (Supplementary figure 2a). In Supplementary figure 2b and 2c we plot the extracted linewidths of P1 and P2 in Fig. 1b in the main text. The linewidth broadens with increasing temperature. We extract the phonon activation energies via fitting to the data in Supplementary figure 2b, 2c via $\gamma(T) = \gamma_0 + \gamma_{ac} * T + \frac{\gamma_{LO}}{e^{\frac{E_{LO}}{k_B T}} - 1}^2$. We yield $E_{LO} = 10.47 \pm 0.56\ meV$ for P2 and $E_{LO} = 10.50 \pm 0.13\ meV$ for P1, which confirms previous results about the phonon activation energy of localized emitters in $WSe_2$. Supplementary figure 2d and 2e depict the integrated counts as a function of temperature for P2 and P1. The red lines are the fit by the function: $I(T) = \frac{I_0}{1 + A_1 e^{\frac{E_1}{k_b T}} + A_2 e^{\frac{E_2}{k_b T}}}$. We could reproduce our experimental data with the characteristic energy $E_1 = 3.67 \pm 0.52\text{meV}$, $E_2 = 20.35 \pm 4.48\text{meV}$ for P2 and $E = 10.402 \pm 1.077\text{meV}$ by taking into account one loss channel for P1. All the extracted energies coincide with former measured results[2]. Supplementary fig. 2f shows the intensity ratio between P1 and P2 as a function of temperature. The intensity of P1 decreases much faster than P2 above 20K.

**Supplementary Note 3: Details of the optical setup**

The sample comprisi3g the $WSe_2$ monolayer is attached to the cold-finger of a liquid Helium flow cryostat (Janis ST-500). A 50x objective (NA=0.42) is used to excite and collect the photoluminescence from the flake. The $WSe_2$ monolayer flake is excited by a Continuous-Wave (CW) 532nm green laser, or a mode-locked pulsed laser, coupled into the beam by a 92:8 pellicle beam splitter. A short-pass filter (670nm) is inserted into the excitation arm. In the collection arm, a 690nm long pass filter was used to cut off the laser and lead the luminescence into the spectrometer (Princeton-Instrument SP2750i), which is equipped with a liquid nitrogen cooled charge coupled detector. To enable the pulse excitation, we used a mode-locked Ti: sapphire laser with the pulse duration of ~3ps, repetition rate of 82MHz and wavelength of ~950nm. The long wavelength pulse is up-converted to high frequency (~475nm) after passing through the frequency converter. A 50:50 beam splitter is used to combine both the green laser and pulse laser beam. For the photon cross-correlation measurements, the emitted photons are passed through a 50:50 beam splitter, where the transmitted photoluminescence is used to select the biexciton emission (~722.3nm). Thanks to the pure spectrum we could directly collect the pure exciton emission after passing the reflected light through a band-pass filter (center 720.6nm) composed of a long pass (720nm) filter and short pass (721nm) filter. The collected photons are coupled into two Silicon-based avalanche photon diodes (APD) with a timing resolution of $t_{\mathrm{Res}} \approx 350\,ps$ and the coincidence events are measured on the time-correlation measurement setup (SPC130). For the autocorrelation measurement, the collected photons (exciton or biexciton) are directly guided to a multi-mode fiber based 50:50 beam splitter and then the second order autocorrelation function $g^2(\tau)$ is measured.

**Supplementary References:**


1. Tonndorf, P. Schmidt, R. *et al.* Single-photon emission from localized excitons in an atomically thin semiconductor. *Optica.* **2,** 347–352 (2015).

2. He, Y. M., *et al.* Phonon induced line broadening and population of the dark exciton in a deeply

trapped localized emitter in monolayer $WSe_2$. *Optics Express.* **24,** 8066–8073 (2016)
