# Supplementary material for: Cascaded emission of single photons from the biexciton in monolayered WSe2
Source: Nat Commun. 2016 Nov 10;7:13409. doi: 10.1038/ncomms13409 (PMC5109589; doi:10.1038/ncomms13409)
Supplement: Peer Review File [file ncomms13409-s2.pdf]

Reviewers' comments:

Reviewer #1 (Remarks to the Author):

The manuscript reports observation of photoluminescence from bound biexcitons in WSe2 at cryogenic temperatures. It shows conclusively that a cascade has been observed between biexciton and single exciton states.

This result is both novel and interesting to the 2D materials and broader quantum photonics communities. Exciton cascades in semiconductor quantum dots grown by the Stranski Krastanow method have led to polarisation-entangled photons in cases where the two orthogonal exciton states are degenerate. Similar physics in 2D materials could lead to novel devices and interesting additional physics.

The data are well presented and of high quality. The analysis of Hanbury-Brown and Twiss datasets is not very transparent though and it would be helpful if the authors could provide the fitting functions used so that readers can judge their veracity.

While the claim of having observed the bound biexciton is well supported, the data presented appear to be from only one defect and no comments are made about the abundance of such observations. This is important to include in order to understand the significance of the work - if it is a 'one off' it is of less value than if all of the bound excitons observed in these samples show accompanying biexciton emission. In previous reports of single photon emission in these materials (eg Koperski et al, ref 10 in the paper), the bound biexciton has been sought but not found, and the authors of the present manuscript argue that their method of depositing the WSe2 flake on an inert GaInP substrate provides improved optical properties and infer that this leads to the appearance of the bound biexciton. Do the authors find this method to be reproducible?

In a similar vein some more detail on sample preparation would help others to reproduce their results - what orientation is the GaInP, how was it grown and what was the surface cleaning recipe?

The work is well referenced and presented in a lucid manner with appropriate abstract introduction and conclusions. There are a few grammatical errors which can be corrected easily enough.

In summary I would recommend publication provided some elaboration is included that will help others to assess the significance of the work and to reproduce the results.

Reviewer #2 (Remarks to the Author):

This work reports the first observation of cascade emission of biexciton and exciton, which is a signature of 0 dimensionally confined quantum dot, from the defect sites of exfoliated WSe2 monolayer. Resolution-limited line width of emission peak and antibunching of emission were demonstrated as evidences of single photon emissions from localized exctions. In addition to above findings, which were already reported from previous literatures, authors convincingly showed the existence of biexcitons with power-dependent and polarized PL measurement and the PL life time measurement. Lastly cross-correlation of sequential exciton and biexciton emissions was measured to show the cascaded emission. Single photon emission from localized exciton of TMD monolayers is of keen interests as a new physical system for single photon source, and since the finding of single photon emission of monolayer WSe2, observation of cascade emission of biexciton and excitons was a question to be answered for some time. This work seems to provide a clear answer to that question and in that regard this work is very timely. The quality of experimental results is high and the analysis and the reasoning argument are also clear. I think

this work merits the publication in Nature Communications.
Only thing that puzzles me is what made it possible for this cascaded emission of biexciton and exciton to be observed? High sample quality due to the use of smooth and inert GaInP was mentioned but the exact origin is not clearly given, such as what is so different from other substrates used in previous works and what natures of GaInP and why they were the key factors. Was the result of other WSe2 samples on different substrate different? More discussion on the effects of GaInP and some case study on the choice of the substrates are recommended.

Reviewer #3 (Remarks to the Author):

In my opinion, this is an important and timely paper, proving that emission from certain localized states in WSe_2 flake have biexciton (XX) character. The localized exciton recombination is studied by time resolved and correlation spectroscopy. Authors selected a few pairs of sharp emission lines and attributed them to consecutive recombination of biexciton and exciton complexes. The arguments leading to such identification are convincing and based on detailed spectroscopic properties such as linearly polarized fine-splitting, super- and supra-linear intensity dependence on the excitation power and the most important - photon cross-correlation. The last, conclusive experiment was feasible thanks to significant improvement of the stability of the WSe_2 monolayers transferred on the epitaxially grown substrates.

The importance of the paper resides in elegant and conclusive evidence of the biexciton nature of some of the single photon sources observed in WSe2 monolayers. The work answers an important question on the origin of the sharp lines, although the precise mechanism of the exciton localization remains unknown. The paper indicates a potential of new discoveries possible for localized emitters. This field could be as reach as field of excitonic complexes in epitaxial semiconductor quantum dots.

Despite of high quality and clarity of the paper I have a few questions and remarks:

- The lowest excitonic state in monolayer WSe_2 is commonly assumed to be a dark state lying several meV below the optically bright state. I would expect that such dark state is present also for the localized excitons. Such state would influence significantly the dynamics of X - XX formation and recombination. It would form a new channel of the XX formation from long-lived dark exciton and would probably enhance biexciton line and suppress X-XX cascade observed in cross correlation experiment. Authors should discuss such possibility.

- The repetition rate of the laser in time resolved experiment was quite high as compared to X lifetime. I would prefer to see complete PL transients (from negative to positive delay) presented in figure 1f. Is there any long PL tail ?

- It would be interesting to compare the excitation power dependencies measured with CW laser to similar curves measured with pulsed excitation. Both experiments should exhibit different saturation behavior and would give additional test of the proposed interpretation.

- The information on the crystallographic orientation of the semiconductor substrate is missing, I would include it.
- The statement "We first prove the quantum nature of our system..." seems to be exaggerated. I can hardly imagine non-quantum photon emission in any semiconductor.

Response to Referee A - Referees's comments quoted in italics

We thank the referee for his report and his supportive comments. The following statements are then made:

The manuscript reports observation of photoluminescence from bound biexcitons in WSe2 at cryogenic temperatures. It shows conclusively that a cascade has been observed between biexciton and single exciton states. This result is both novel and interesting to the 2D materials and broader quantum photonics communities. Exciton cascades in semiconductor quantum dots grown by the Stranski Krastanow method have led to polarisation-entangled photons in cases where the two orthogonal exciton states are degenerate. Similar physics in 2D materials could lead to novel devices and interesting additional physics. The data are well presented and of high quality. The analysis of Hanbury-Brown and Twiss datasets is not very transparent though and it would be helpful if the authors could provide the fitting functions used so that readers can judge their veracity.

We thank the referee for carefully reading our manuscript and pointing this out. We have widely expanded the methods section of the manuscript, and hope that now things are clearer.

While the claim of having observed the bound biexciton is well supported, the data presented appear to be from only one defect and no comments are made about the abundance of such observations. This is important to include in order to understand the significance of the work - if it is a 'one off' it is of less value than if all of the bound excitons observed in these samples show accompanying biexciton emission. In previous reports of single photon emission in these materials (eg Koperski et al, ref 10 in the paper), the bound biexciton has been sought but not found, and the authors of the present manuscript argue that their method of depositing the WSe2 flake on an inert GaInP substrate provides improved optical properties and infer that this leads to the appearance of the bound biexciton. Do the authors find this method to be reproducible?

In fact, we were also wondering if biexcitons appear reproducibly in our sample. We thus took the effort, and searched for more pairs, with success. Supplementary Fig.S5 lists another biexciton emission from the same wafer. This observation supports the reproducibility of the method.

In a similar vein some more detail on sample preparation would help others to reproduce their results - what orientation is the GaInP, how was it grown and what was the surface cleaning recipe?

We would like to thank the referee for pointing this out. We rewrote the methods part and it's more explicit now.

The work is well referenced and presented in a lucid manner with appropriate abstract introduction and conclusions. There are a few grammatical errors which can be corrected easily enough. In summary I would recommend publication provided some elaboration is included that will help others to assess the significance of the work and to reproduce the results.

We would like to thank the referee once again for his comments and we hope that we have corrected the English in the current version of the manuscript.

Response to Referee B - Referees's comments quoted in italics

We thank the referee for his detailed report. He states:

This work reports the first observation of cascade emission of biexciton and exciton, which is a signature of 0 dimensionally confined quantum dot, from the defect sites of exfoliated WSe2 monolayer. Resolution-limited line width of emission peak and antibunching of emission were demonstrated as evidences of single photon emissions from localized exctions. In addition to above findings, which were already reported from previous literatures, authors convincingly showed the existence of biexcitons with power-dependent and polarized PL measurement and the PL life time measurement. Lastly cross-correlation of sequential exciton and biexciton emissions was measured to show the cascaded emission. Single photon emission from localized exciton of TMD monolayers is of keen interests as a new physical system for single photon source, and since the finding of single photon emission of monolayer WSe2, observation of cascade emission of biexciton and excitons was a question to be answered for some time. This work seems to provide a clear answer to that question and in that regard this work is very timely. The quality of experimental results is high and the analysis and the reasoning argument are also clear. I think this work merits the publication in Nature Communications. Only thing that puzzles me is what made it possible for this cascaded emission of biexciton and exciton to be observed?

We thank the referee for his overall very positive assessment. We have implemented an additional discussion in the mainbody text prior to the conclusion to assess on the referee's point: A detailed understanding of the microscopic properties of the localized biexciton and its optical selection rules in monolayers of WSe2 require more work for clarification. The free biexciton states in WSe2 monolayers have been predicted by the variational calculation, and experimental indications were discussed previously (see You,Y. etc. Nature. Physics. 11, 477481 (2015). Similarily to the expectations for free biexcitonic states in WSe2, our lifetime measurements indicate that the localized biexcitons are probably larger than the exciton.

In fact, we believe that our results are not surprising, it is rather surprising that this observation has not been made earlier by other groups which have observed excitonic features. Even tho this is a bit speculative, we can state (and have stated in the original manuscript), that utilizing GaInP as a substrate notably improves the optical quality of the emitters. A discussion on this topic can be found in the revised supplement (Fig S4). It reads: The successful observation of the localized biexciton in our work is a consequence of the very clean spectra, which in turn are a result of our hybrid semiconductor-monolayer heterostructure. To investigate the emission properties of the localized excitons in WSe2 monolayers on different substrates, Figure S4(a) and S4(b) depict the photoluminescence from two monolayer WSe2, transferred on a SiO2/Si and a GaInP/GaAs substrate. The observed linewidth for GaInP substrate is more than one order of magnitude narrower than that for SiO2 substrate. One hypothesis is the narrowing due to the fewer charge fluctuation for non-insulating environment which allows transferring trapped charges, which leads to smaller spectral jitter. In addition, recent localized exciton studies on the influence of the substrate have revealed that the localized exciton linewidth and spectral wandering would be significantly enhanced after removing the SiO2 substrate.

High sample quality due to the use of smooth and inert GaInP was mentioned but the exact origin is not clearly given, such as what is so different from other substrates used in

previous works and what natures of GaInP and why they were the key factors. Was the result of other WSe2 samples on different substrate different? More discussion on the effects of GaInP and some case study on the choice of the substrates are recommended.

We would like to thank the referee for pointing this out. We rewrote one passage to discuss the effects of GaInP substrate, as stated above.

Response to Referee C - Referees's comments quoted in italics

We thank the referee for his report and his supportive comments. The referee gives a clear summary of the manuscript, and comments on the interest and timeliness of our results. The following statements are then made:

In my opinion, this is an important and timely paper, proving that emission from certain localized states in WSe_2 flake have biexciton (XX) character. The localized exciton recombination is studied by time resolved and correlation spectroscopy. Authors selected a few pairs of sharp emission lines and attributed them to consecutive recombination of biexciton and exciton complexes. The arguments leading to such identification are convincing and based on detailed spectroscopic properties such as linearly polarized fine-splitting, super- and supra-linear intensity dependence on the excitation power and the most important - photon cross-correlation. The last, conclusive experiment was feasible thanks to significant improvement of the stability of the WSe_2 monolayers transferred on the epitaxially grown substrates. The importance of the paper resides in elegant and conclusive evidence of the biexciton nature of some of the single photon sources observed in WSe2 monolayers. The work answers an important question on the origin of the sharp lines, although the precise mechanism of the exciton localization remains unknown. The paper indicates a potential of new discoveries possible for localized emitters. This field could be as reach as field of excitonic complexes in epitaxial semiconductor quantum dots. Despite of high quality and clarity of the paper I have a few questions and remarks:
- The lowest excitonic state in monolayer WSe_2 is commonly assumed to be a dark state lying several meV below the optically bright state. I would expect that such dark state is present also for the localized excitons. Such state would influence significantly the dynamics of X - XX formation and recombination. It would form a new channel of the XX formation from long-lived dark exciton and would probably enhance biexciton line and suppress X-XX cascade observed in cross correlation experiment. Authors should discuss such possibility.

We agree with the referee about the possibility of an interplay of a dark exciton with the XX-X cascade. In fact, in a conventional InAs QD, the presence of a dark state which contributes to the emission leads to a long time bunching behavior in the exciton correlation (see, e.g. A. Ulhaq et. al. Nat. Photonics 6, 238 (2012)). Our CW correlation measurements in the supplement do not show any signature of a long time bunching superimposing the observed antibunching. This leads us to the conclusion, in agreement with our previous observations in (He et al. Optics Experss (2016)), that the dark state is sparsely populated at lowest temperatures. It will be interesting to study, whether a temperature induced population of the biexciton channel will lead to a significant modification of the quantum statistics in our observed cascade. The corresponding passage which now reads:
The microscopic properties of the localized biexciton and its selection rules in monolayers of WSe2 require more work for clarification. The free biexciton states in WSe2 monolayers have been predicted by variational calculations and experimental indications were previously

discussed28. Similarily to the expectations for free biexcitonic states in WSe2, our lifetime measurements indicate that the localized biexcitons are probably larger than the exciton. Furthermore, a previous study of localized excitons in WSe2 indicated the presence of a dark localized exciton as well as a free dark exciton state several meV below the bright exciton37,38. In our current study, we did not observe any significant contribution of a dark state in the chosen temperature range, which would necessarily lead to a blinking in the defect emission, and thus to a long term bunching in the autocorrelation of the exciton emission. However, it will be interesting to see, if e.g. a temperature induced population of the dark exciton will lead to a modification of the observed quantum statistics in our cascaded system.

- The repetition rate of the laser in time resolved experiment was quite high as compared to X lifetime. I would prefer to see complete PL transients (from negative to positive delay) presented in figure 1f. Is there any long PL tail ?

We thank the referee for carefully reading our manuscript. To avoid any misunderstanding, we have additionally included normalized the curves corresponding to the data set presented in the figure S6 in the supplement and extended the timescale to one laser period(12.2ns) . Compared to the XX, the X line was weak under higher excitation power and we took longer time to perform the lifetime measurement. Thus, the contribution of the APD (Avalanche Photon Diode) dark counts for X lifetime measurement would be higher and we could see slightly high offset. Furthermore, the data for X in figure 1f didn't show obvious long PL tail.

It would be interesting to compare the excitation power dependencies measured with CW laser to similar curves measured with pulsed excitation. Both experiments should exhibit different saturation behavior and would give additional test of the proposed interpretation.

We agree with the referee that the pulsed and CW excitation could produce very different exciton populations. The population of multiexcitons would scale differently with power and such behavior would shed more light into the fluorescence dynamics. Detailed studies about the saturation behavior, fluorescence blinking statistics, lifetime dynamics,spectral wandering under the impact of pulsed versus cw excitation source would help clarify the multiexcitonic dynamics of the emitters. However, we believe that such a demonstration is beyond the scope of the paper, as the primary target of this work is the demonstration of a localized biexciton, and not a detailed study of its dynamics.

The information on the crystallographic orientation of the semiconductor substrate is missing, I would include it.

We would like to thank the referee for pointing this out. We rewrote the methods part and it's more explicit now.

The statement We first prove the quantum nature of our system... seems to be exaggerated. I can hardly imagine non-quantum photon emission in any semiconductor.

In order to avoid the misunderstanding, we rewrote the corresponding sentence which now reads:
We prove the capability of our system to emit quantum light by measuring the second order autocorrelation function of the emission feature P2 under pulse-laser excitation, which we have

attributed to the excitonic state.

REVIEWERS' COMMENTS:

Reviewer #1 (Remarks to the Author):

I am content that the authors have addressed all my previous comments, and recommend the paper for publication.

Reviewer #2 (Remarks to the Author):

All issues I raised were addressed well in the revised version. I support the publication of this paper in Nature Communications as it is.

Reviewer #3 (Remarks to the Author):

I appreciate the explanations given by authors. In my opinion the answers and corrections in the manuscript are sufficient. Even if the role of the dark exciton is not yet clear I believe that the results are important, timely and deserve publication. Therefore I am convinced that the paper is suitable for publication in the present form.
